# Endoscopic ultrasound-guided pancreaticogastrostomy using lumen-apposing metal stent to manage a case of pseudocyst and pancreatic duct dilation after severe acute pancreatitis

**DOI:** 10.1055/a-2830-4108

**Published:** 2026-04-02

**Authors:** Yujie Guo, Yuqin Jin, Hengchao Li, Deliang Fu, Chen Jin, Liang Zhong

**Affiliations:** 1 159397 Department of Pancreatic Surgery, Huashan Hospital Fudan University, Shanghai, China; 2 159397 Department of Gastroenterology and Endoscopy, Huashan Hospital Fudan University, Shanghai, China

Severe acute pancreatitis frequently induces pancreatic necrosis and pancreatic duct disruption, leading to the development of pseudocysts. The majority of these pseudocysts maintains direct communication with the disrupted duct. For such cases, endoscopic retrograde pancreatic drainage (ERPD) typically serves as the primary treatment 1 . However, pancreatic duct discontinuity—resulting from duct rupture, cyst compression or fibrotic tissue proliferation—can render ERPD formidable. Consequently, the management of these patients poses a significant clinical challenge.

Traditionally, such patients required surgical intervention; however, with the advancement of endoscopic techniques, particularly the emergence of endoscopic ultrasound (EUS)-guided pancreaticogastrostomy, many patients can now be treated with a minimally invasive approach. However, this procedure is not only technically challenging but also the choice of the stent still remains controversial. While the use of lumen-apposing metal stents (LAMSs) for endoscopic management of pancreatic fluid collections (PFCs) is increasing 2 , their application in pancreaticogastrostomy is exceedingly rare 3 4 5 .

**Fig. 1:**
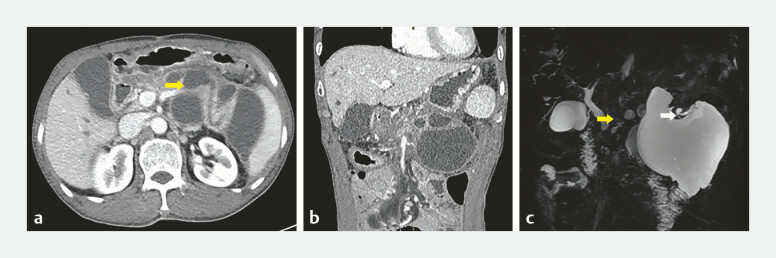
Preoperative abdominal CT and MRCP. **a** Pancreatic duct dilation with pseudocyst formation (yellow arrow: pancreatic duct). **b** Dilated pancreatic duct abuts the gastric wall. **c** Proximal pancreatic duct discontinuity (yellow arrow) with distal duct rupture communicating with a pseudocyst (white arrow). CT, computed tomography; MRCP, magnetic resonance cholangiopancreatography.

A 53-year-old man was referred to our unit with a history of severe necrotizing pancreatitis complicated by a pancreatic duct dilation and pseudocyst ( Fig. 1 ); ERPD failed because the guidewire could not pass through the obstructed pancreatic duct at the neck of pancreas; EUS-guided cystogastrostomy failed because the distance between the cyst wall and the gastric wall was more than 3 cm. EUS-guided pancreaticogastrostomy was successfully performed using a LAMS ( Video 1 ).

Endoscopic ultrasound-guided pancreaticogastrostomy using a lumen-apposing metal stent to manage a case of pseudocyst and pancreatic duct dilation after severe acute pancreatitis.Video 1

**Fig. 2:**
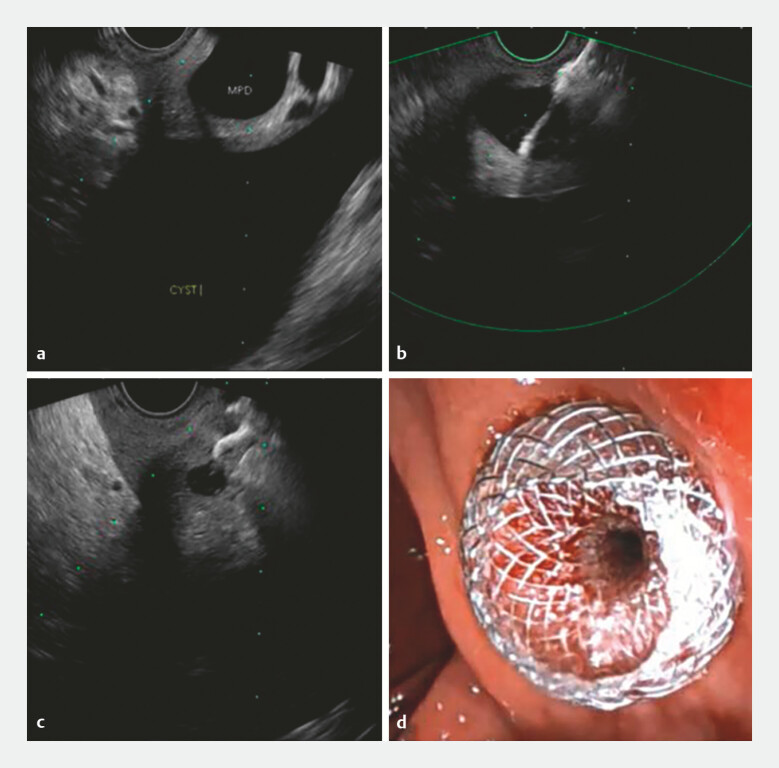
EUS-guided dilated pancreatic duct access and LAMS placement. **a** EUS revealed the dilated pancreatic duct. **b** Puncture of the pancreatic duct. **c** LAMS deployment under EUS. **d** LAMS deployment under endoscopy. EUS, endoscopic ultrasound; LAMS, lumen-apposing metal stent.

**Fig. 3:**
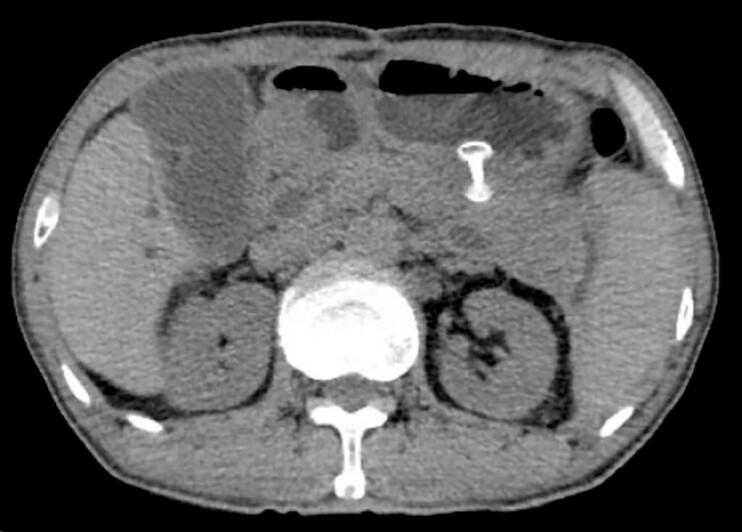
Postoperative abdominal computed tomography shows the resolution of pseudocyst and pancreatic duct dilation.

The procedure began with (EUS) to localize the dilated pancreatic duct (reach a diameter of 23 mm). Under ultrasonic guidance, a Boston Scientific Hot AXIOS LAMS (8 mm × 8 mm) was punctured into the pancreatic duct ( Fig. 2 ). The procedure was completed in 16 minutes, and the patient had no postoperative complications. Follow-up abdominal computed tomographic (CT) scan on postoperative day 2 revealed a significant reduction in the size of the pseudocyst, with the pancreatic duct no longer dilated. A repeat CT scan at the 1-month postoperative follow-up demonstrated near-complete resolution of the pseudocyst and pancreatic duct dilation ( Fig. 3 ).

For patients presenting with both a pseudocyst and a dilated pancreatic duct, where the dilated duct communicates directly with the pseudocyst, EUS-guided pancreaticogastrostomy using the LAMS may be a safe and effective option if the dilated duct could contain distal flange and the distance between the pancreatic duct and the gastric wall is less than 1 cm. However, the choice of stent type requires further clinical investigation.

Endoscopy_UCTN_Code_TTT_1AS_2AI

Endoscopy_UCTN_Code_TTT_1AS_2AJ
